# Supplementary material for: Flexible-type ultrathin holographic endoscope for microscopic imaging of unstained biological tissues
Source: Nat Commun. 2022 Aug 2;13:4469. doi: 10.1038/s41467-022-32114-5 (PMC9345988; doi:10.1038/s41467-022-32114-5)
Supplement: Supplementary file 1 — Supplementary Information [file 41467_2022_32114_MOESM1_ESM.pdf]

Flexible-type ultrathin holographic endoscope for microscopic imaging of unstained biological tissues

Wonjun Choi[1,2,+], Munkyu Kang[1,2,+], Jin Hee Hong[1,2], Ori Katz[3], Byunghak Lee[4,5], Guang Hoon Kim[4], Youngwoon Choi[6,7,*], and Wonshik Choi[1,2,*]

[1]*Center for Molecular Spectroscopy and Dynamics, Institute for Basic Science (IBS), Seoul 02841, Republic of Korea.*
[2]*Department of Physics, Korea University, Seoul 02841, Republic of Korea*
[3]*Department of Applied Physics, The Selim and Rachel Benin School of Computer Science & Engineering, The Hebrew University of Jerusalem, Jerusalem 9190401, Israel*
[4]*Korea Electrotechnology Research Institute, Ansan, 15588, Korea*
[5]*B2LAB co., ltd., 37673, Korea*
[6]*Department of Bioengineering, Korea University, Seoul 02841, Republic of Korea*
[7]*Interdisciplinary Program in Precision Public Health, Korea University, Seoul 02841, Republic of Korea*
[+]*These authors contributed equally to this work.*
[*]*e-mail: youngwoon@korea.ac.kr and wonshik@korea.ac.kr*

1. Image formation in the lensless configuration

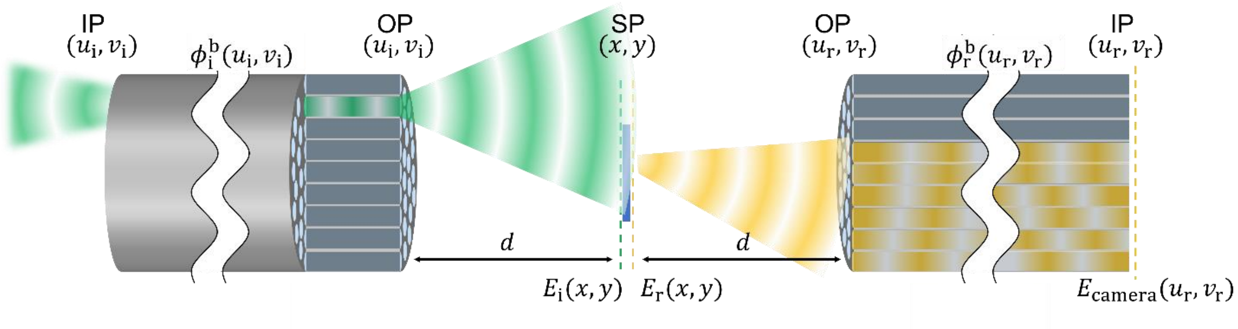

Supplementary Figure 1. Detailed layout of the image formation process.

The theoretical details about the image formation in our endomicroscope are presented in this section (Supplementary Figure 1). A focused beam illuminating a single core located at (u_i, v_i) on IP, the proximal end of the fibre bundle, travels through the same core (u_i, v_i) on OP, the distal end of the fibre bundle. And the beam further propagates through free space as a parabolic wave arriving at (x, y) on SP, the sample plane. The incident wave at SP is represented as:

$$E_i(x, y; u_i, v_i) = \frac{e^{ikd}}{i\lambda d} exp\left\{i\frac{k}{2d}[(x-u_i)^2 + (y-v_i)^2]\right\} e^{i\phi_i^b(u_i,v_i)}. \quad \text{(S1)}$$

Here $\phi_i^b(u_i, v_i)$ is the phase retardation of the incident wave imposed by the fibre core at (u_i, v_i), and $k = 2\pi/\lambda$. The reflected wave from the target object is given as

$$E_r(x, y; u_i, v_i) = O(x, y)E_i(x, y; u_i, v_i), \quad \text{(S2)}$$

where $O(x, y)$ is the object function. This reflected wave propagates back to the distal end of the fibre bundle and is picked up by multiple fibre cores. The wave picked up by a core located at (u_r, v_r) on the OP is described by

$$E_o(u_r, v_r; u_i, v_i) = \frac{e^{ikd}}{i\lambda d} \iint E_r(x, y; u_i, v_i) exp\left\{i\frac{k}{2d}[(u_r-x)^2 + (v_r-y)^2]\right\} dxdy. \quad \text{(S3)}$$

By inserting Eq. (S2) into Eq. (S3), we obtain the representation of the reflected wave at OP as

$$E_{\mathrm{o}}(u_{\mathrm{r}},v_{\mathrm{r}};u_{\mathrm{i}},v_{\mathrm{i}}) = -\frac{e^{2ikd}}{\lambda^2 d^2} exp\left\{i\frac{k}{2d}({u_{\mathrm{i}}}^2+{v_{\mathrm{i}}}^2)+i\phi_{\mathrm{i}}^{\mathrm{b}}(u_{\mathrm{i}},v_{\mathrm{i}})\right\} \times \left\{i\frac{k}{2d}(u_{\mathrm{r}}^2+v_{\mathrm{r}}^2)\right\}$$
$$\times \iint O(x,y) exp\left\{i\frac{k}{d}(x^2+y^2)\right\} exp\left\{-i\frac{k}{d}[(u_{\mathrm{r}}+u_{\mathrm{i}})x+(v_{\mathrm{r}}+v_{\mathrm{i}})y]\right\} dxdy. \quad \text{(S4)}$$

When this wave travels back through the fibre core and reaches the proximal end of the core at IP, an additional phase retardation $\phi_{\mathrm{r}}^{\mathrm{b}}(u_{\mathrm{r}},v_{\mathrm{r}})$ occurs in the wave at the fibre core $(u_{\mathrm{r}},v_{\mathrm{r}})$. Therefore, the wave observed at $(u_{\mathrm{r}},v_{\mathrm{r}})$ on IP is given as

$$E_{\mathrm{camera}}(u_{\mathrm{r}},v_{\mathrm{r}};u_{\mathrm{i}},v_{\mathrm{i}}) = E_{\mathrm{o}}(u_{\mathrm{r}},v_{\mathrm{r}};u_{\mathrm{i}},v_{\mathrm{i}}) \times e^{i\phi_{\mathrm{r}}^{\mathrm{b}}(u_{\mathrm{r}},v_{\mathrm{r}})}$$
$$= -\frac{e^{2ikd}}{\lambda^2 d^2} exp\left\{i\frac{k}{2d}({u_{\mathrm{i}}}^2+{v_{\mathrm{i}}}^2)+i\phi_{\mathrm{i}}^{\mathrm{b}}(u_{\mathrm{i}},v_{\mathrm{i}})\right\} \times exp\left\{i\frac{k}{2d}(u_{\mathrm{r}}^2+v_{\mathrm{r}}^2)+i\phi_{\mathrm{r}}^{\mathrm{b}}(u_{\mathrm{r}},v_{\mathrm{r}})\right\} \times$$
$$\iint O(x,y) exp\left\{i\frac{k}{d}(x^2+y^2)\right\} exp\left\{-i\frac{k}{d}[(u_{\mathrm{r}}+u_{\mathrm{i}})x+(v_{\mathrm{r}}+v_{\mathrm{i}})y]\right\} dxdy. \quad \text{(S5)}$$

In Eq. (S5), the expression $O(x,y) exp\left\{i\frac{k}{d}(x^2+y^2)\right\} \equiv O_{\mathrm{M}}(x,y)$ in the double integral can be considered a modified object function as it is invariant with respect to the choice of illumination core as well as detection core. The integral in Eq. (S5) corresponds to the Fourier transform of $O_{\mathrm{M}}(x,y)$, i.e. $\tilde{O}_{\mathrm{M}}(k_x,k_y)$, with $k_x = \frac{k}{d}(u_{\mathrm{r}}+u_{\mathrm{i}})$ and $k_y = \frac{k}{d}(v_{\mathrm{r}}+v_{\mathrm{i}})$. Therefore, $(u_{\mathrm{r}},v_{\mathrm{r}})$ at OP is the Fourier plane of the object function with the scaling factor k/d and the spectral shift associated with the position of the illumination core $(u_{\mathrm{i}},v_{\mathrm{i}})$. The phase term in the first exponential in Eq. (S5), $\frac{k}{2d}({u_{\mathrm{i}}}^2+{v_{\mathrm{i}}}^2)+\phi_{\mathrm{i}}^{\mathrm{b}}(u_{\mathrm{i}},v_{\mathrm{i}}) \equiv \phi_{\mathrm{i}}(u_{\mathrm{i}},v_{\mathrm{i}})$, is an addition of the quadratic phase due to the Fresnel diffraction and the phase retardation imposed by the fibre bundle. Altogether this term can be considered as the input aberration occurring in the illumination beam path. Similarly, $\frac{k}{2d}(u_{\mathrm{r}}^2+v_{\mathrm{r}}^2)+\phi_{\mathrm{r}}^{\mathrm{b}}(u_{\mathrm{r}},v_{\mathrm{r}}) \equiv \phi_{\mathrm{r}}(u_{\mathrm{r}},v_{\mathrm{r}})$ can be considered as the output aberration occurring in the detection beam path. After all these considerations, Eq. (S5) is simplified as

$$E_{\mathrm{camera}}(u_{\mathrm{r}},v_{\mathrm{r}};u_{\mathrm{i}},v_{\mathrm{i}}) = -\frac{e^{2ikd}}{\lambda^2 d^2} e^{i\phi_{\mathrm{i}}(u_{\mathrm{i}},v_{\mathrm{i}})} \tilde{O}_{\mathrm{M}}\left(\frac{k}{d}(u_{\mathrm{r}}+u_{\mathrm{i}}),\frac{k}{d}(v_{\mathrm{r}}+v_{\mathrm{i}})\right) e^{i\phi_{\mathrm{r}}(u_{\mathrm{r}},v_{\mathrm{r}})}. \quad \text{(S6)}$$

This is formally equivalent to the general imaging case for an object at the focal plane of an objective lens except for the constant factor, $-e^{2ikd}\lambda^{-2}d^{-2}$. Notably, the CLASS algorithm can find and correct the quadratic phases induced by the Fresnel diffractions in ϕ_{i} and ϕ_{r}, because it is equivalent to the automatic numerical refocusing. Thus, the CLASS algorithm itself acts as a numerical objective lens. Furthermore, the depth information d of the target object can be extracted by the term $exp\left\{i\frac{k}{d}(x^2+y^2)\right\}$ in the modified object function $O_{\mathrm{M}}(x,y)$. This adds the 3D imaging capability to our endoscope.

2. Details of CLASS algorithm for the lensless endoscope

Our CLASS algorithm consists of two-step processes. One is the forward process that preferably corrects the input phase retardation $\phi_{\mathrm{i}}(u_{\mathrm{i}}, v_{\mathrm{i}})$, and the other is the correction in the backward process that resolves the output phase retardation $\phi_{\mathrm{r}}(u_{\mathrm{r}}, v_{\mathrm{r}})$. Here, we describe each process in detail.

(i) Forward correction process

The representative interference images recorded at the camera are shown in Supplementary Figure 2a. The strong back-reflection noise is confined only within the illumination cores, which is seen as the bright spots in Supplementary Figure 2a, while the sample information is distributed through the other cores. By taking Hilbert transforms of the raw interferograms, we obtained the complex field images $E_{\mathrm{camera}}(u_{\mathrm{r}}, v_{\mathrm{r}}; u_{\mathrm{i}}, v_{\mathrm{i}})$. The corresponding amplitude maps of E_{camera} are shown in Supplementary Figure 2a. To find the input aberration $\phi_{\mathrm{i}}(u_{\mathrm{i}}, v_{\mathrm{i}})$, we compensated the spectral shift of E_{camera} due to the scanning of the illumination core $(u_{\mathrm{i}}, v_{\mathrm{i}})$. This was done by converting $E_{\mathrm{camera}}(u_{\mathrm{r}}, v_{\mathrm{r}}; u_{\mathrm{i}}, v_{\mathrm{i}})$ to $E_{\mathrm{camera}}(u_{\mathrm{r}} - u_{\mathrm{i}}, v_{\mathrm{r}} - v_{\mathrm{i}}; u_{\mathrm{i}}, v_{\mathrm{i}})$, the results are shown in Supplementary Figure 2c. Mathematically, the shifted field is written as

$$E_{\mathrm{camera}}(u_{\mathrm{r}} - u_{\mathrm{i}}, v_{\mathrm{r}} - v_{\mathrm{i}}; u_{\mathrm{i}}, v_{\mathrm{i}}) = -\frac{e^{2ikd}}{\lambda^2 d^2} e^{i\phi_{\mathrm{r}}(u_{\mathrm{r}} - u_{\mathrm{i}}, v_{\mathrm{r}} - v_{\mathrm{i}})} \tilde{O}_{\mathrm{M}}\left(\frac{k}{d}u_{\mathrm{r}}, \frac{k}{d}v_{\mathrm{r}}\right) e^{i\phi_{\mathrm{i}}(u_{\mathrm{i}}, v_{\mathrm{i}})}. \quad \text{(S7)}$$

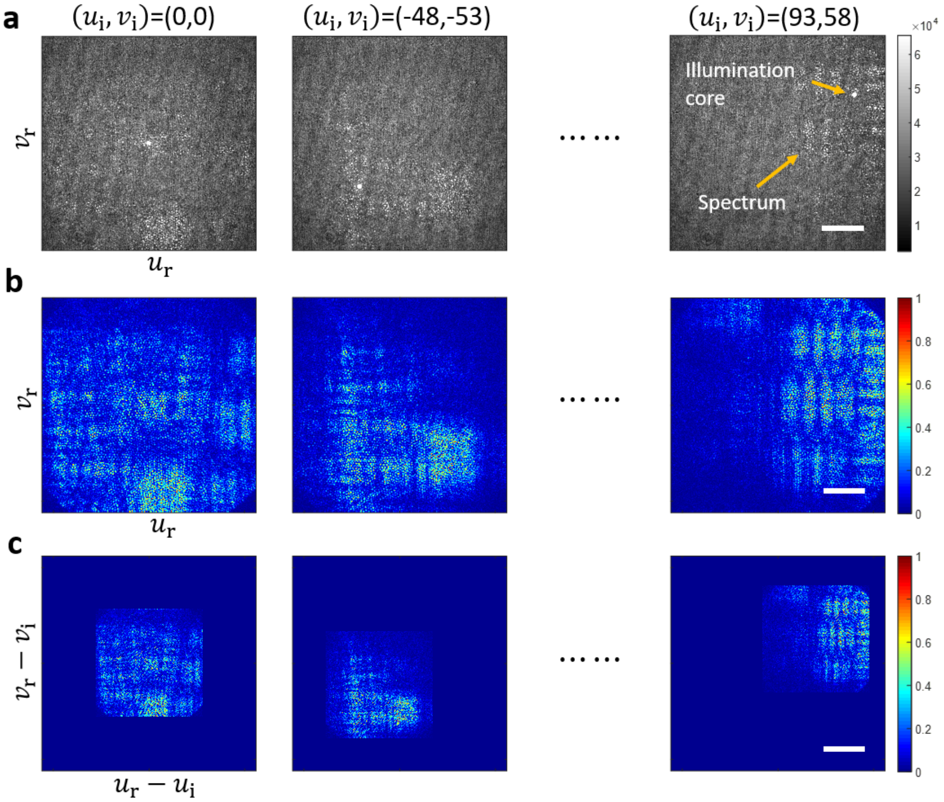

Supplementary Figure 2. Correction in the forward process. a, Raw interference images taken by the camera with the scanning of the illumination cores. The coordinates above each Figure indicate $(u_{\mathrm{i}}, v_{\mathrm{i}})$ in μm. Colour bar, intensity in arbitrary unit. **b,** Amplitude of complex field maps $E_{\mathrm{camera}}(u_{\mathrm{r}}, v_{\mathrm{r}}; u_{\mathrm{i}}, v_{\mathrm{i}})$ for the images in **a**. **c,** Amplitude of complex field maps $E_{\mathrm{camera}}(u_{\mathrm{r}} - u_{\mathrm{i}}, v_{\mathrm{r}} - v_{\mathrm{i}}; u_{\mathrm{i}}, v_{\mathrm{i}})$ after the spectral shift of the images in **b**. Scale bar in **a** and **b** is 50 μm and scale bar in **c** is 100 μm. Colour bar in **b** and **c**, normalized amplitude.

If there were no input and output phase retardations, i.e. $\phi_{\mathrm{i}} = 0$ and $\phi_{\mathrm{r}} = 0$, $E_{\mathrm{camera}}(u_{\mathrm{r}} - u_{\mathrm{i}}, v_{\mathrm{r}} - v_{\mathrm{i}}; u_{\mathrm{i}}, v_{\mathrm{i}})$ becomes identical to the modified object spectrum $\tilde{O}_{\mathrm{M}}$ regardless of the choice of the illumination

core $(u_\mathrm{i}, v_\mathrm{i})$, aside from the constant factor. In the presence of input phase retardations, $E_\mathrm{camera}(u_\mathrm{r} - u_\mathrm{i}, v_\mathrm{r} - v_\mathrm{i}; u_\mathrm{i}, v_\mathrm{i})$ experiences phase shift $\phi_\mathrm{i}(u_\mathrm{i}, v_\mathrm{i})$ depending on the illumination core. Therefore, we computed the correlation between the images in Supplementary Figure 2c to find their relative phase $\phi_\mathrm{i}^{(1)}(u_\mathrm{i}, v_\mathrm{i})$, which is a good approximation of $\phi_\mathrm{i}(u_\mathrm{i}, v_\mathrm{i})$. In fact, $\phi_\mathrm{i}^{(1)}$ is not identical to ϕ_i due to the presence of the output phase retardation $\phi_\mathrm{r}(u_\mathrm{r} - u_\mathrm{i}, v_\mathrm{r} - v_\mathrm{i})$. However, if $\phi_\mathrm{r}(u_\mathrm{r}, v_\mathrm{r})$ is a slowly varying function of $(u_\mathrm{r}, v_\mathrm{r})$, then the difference between $\phi_\mathrm{i}^{(1)}(u_\mathrm{i}, v_\mathrm{i})$ and $\phi_\mathrm{i}(u_\mathrm{i}, v_\mathrm{i})$ is finite enough that the convergence is guaranteed with the iterations explained in the following. In fact, our iteration algorithm ensures the convergence even when $\phi_\mathrm{r}(u_\mathrm{r}, v_\mathrm{r})$ is completely random function of $(u_\mathrm{r}, v_\mathrm{r})$ due to multiple rounds of iterations. We first applied the conjugate of $\phi_\mathrm{i}^{(1)}$ to the measured field for the correction as follows;

$$E_\mathrm{camera}^{(1,\mathrm{F})}(u_\mathrm{r}, v_\mathrm{r}; u_\mathrm{i}, v_\mathrm{i}) = e^{-i\phi_\mathrm{i}^{(1)}(u_\mathrm{i}, v_\mathrm{i})} E_\mathrm{camera}(u_\mathrm{r}, v_\mathrm{r}; u_\mathrm{i}, v_\mathrm{i}). \tag{S8}$$

Here, the superscript $(1, \mathrm{F})$ indicates the first iteration for the forward process.

(ii) Backward correction process

To find ϕ_r, we consider the backward process where the illumination is sent in the reverse direction through $(u_\mathrm{r}, v_\mathrm{r})$. Computationally, this can be done as follows. We construct a reflection matrix R by assigning each pixel of $E_\mathrm{camera}(u_\mathrm{r}, v_\mathrm{r}; u_\mathrm{i}, v_\mathrm{i})$ to a matrix element in a way that the indices for a column and a row correspond to $(u_\mathrm{i}, v_\mathrm{i})$ and $(u_\mathrm{r}, v_\mathrm{r})$, respectively (Supplementary Figure 3a). In matrix R, each column corresponds to the measured image with the illumination core $(u_\mathrm{i}, v_\mathrm{i})$. We then compute its transpose, R_{tr}, describing the backward process (Supplementary Figure 3b). Each column of R_{tr} means the complex field image $E_\mathrm{camera}(u_\mathrm{i}, v_\mathrm{i}; u_\mathrm{r}, v_\mathrm{r})$ that would be recorded by the camera if the illumination was made through the core at $(u_\mathrm{r}, v_\mathrm{r})$.

The backward correction process is added to the preceding forward correction process. This is done as follows. The reflection matrix R is first constructed from E_camera. Let's denote this original matrix R as $R^{(1)}$. The correction for the forward process is applied to $R^{(1)}$ by multiplying the phase correction factor $e^{-i\phi_\mathrm{i}^{(1)}(u_\mathrm{i}, v_\mathrm{i})}$ to each $(u_\mathrm{i}, v_\mathrm{i})$ column, as indicated in Eq. (S8). Then the conjugation matrix $R_{tr}^{(1)}$ is constructed by taking the transpose of this corrected matrix. The representative amplitude maps, $e^{-i\phi_\mathrm{i}^{(1)}(u_\mathrm{i}, v_\mathrm{i})} E_\mathrm{camera}(u_\mathrm{i}, v_\mathrm{i}; u_\mathrm{r}, v_\mathrm{r})$ obtained from the columns of $R_{tr}^{(1)}$ are shown in Supplementary Figure 3c. Similar to the forward process, we applied the spectral shifts to these images to obtain the complex field maps $e^{-i\phi_\mathrm{i}^{(1)}(u_\mathrm{i} - u_\mathrm{r}, v_\mathrm{i} - v_\mathrm{r})} E_\mathrm{camera}(u_\mathrm{i} - u_\mathrm{r}, v_\mathrm{i} - v_\mathrm{r}; u_\mathrm{r}, v_\mathrm{r})$ (Supplementary Figure 3d). Then we can find the approximate output aberration $\phi_\mathrm{r}^{(1)}$ from the correlations among the shifted fields. The correction $\phi_\mathrm{r}^{(1)}$ is applied to the column of $R_{tr}^{(1)}$, and its transpose is taken to obtain the reflection matrix $R^{(2)}$ in the original basis. As the input and output aberrations are corrected, the matrix element is given as

$$E_\mathrm{camera}^\mathrm{c} = -\frac{e^{2ikd}}{\lambda^2 d^2} e^{i\left(\phi_\mathrm{r}(u_\mathrm{r}, v_\mathrm{r}) - \phi_\mathrm{r}^{(1)}(u_\mathrm{r}, v_\mathrm{r})\right)} \tilde{O}_\mathrm{M}\left(\frac{k}{d}(u_\mathrm{r} + u_\mathrm{i}), \frac{k}{d}(v_\mathrm{r} + v_\mathrm{i})\right) e^{i\left(\phi_\mathrm{i}(u_\mathrm{i}, v_\mathrm{i}) - \phi_\mathrm{i}^{(1)}(u_\mathrm{i}, v_\mathrm{i})\right)}. \tag{S9}$$

$\left|\phi_\mathrm{r} - \phi_\mathrm{r}^{(1)}\right|$ tends to be smaller than $\left|\phi_\mathrm{i} - \phi_\mathrm{i}^{(1)}\right|$ because the input aberration is corrected prior to the output aberration.

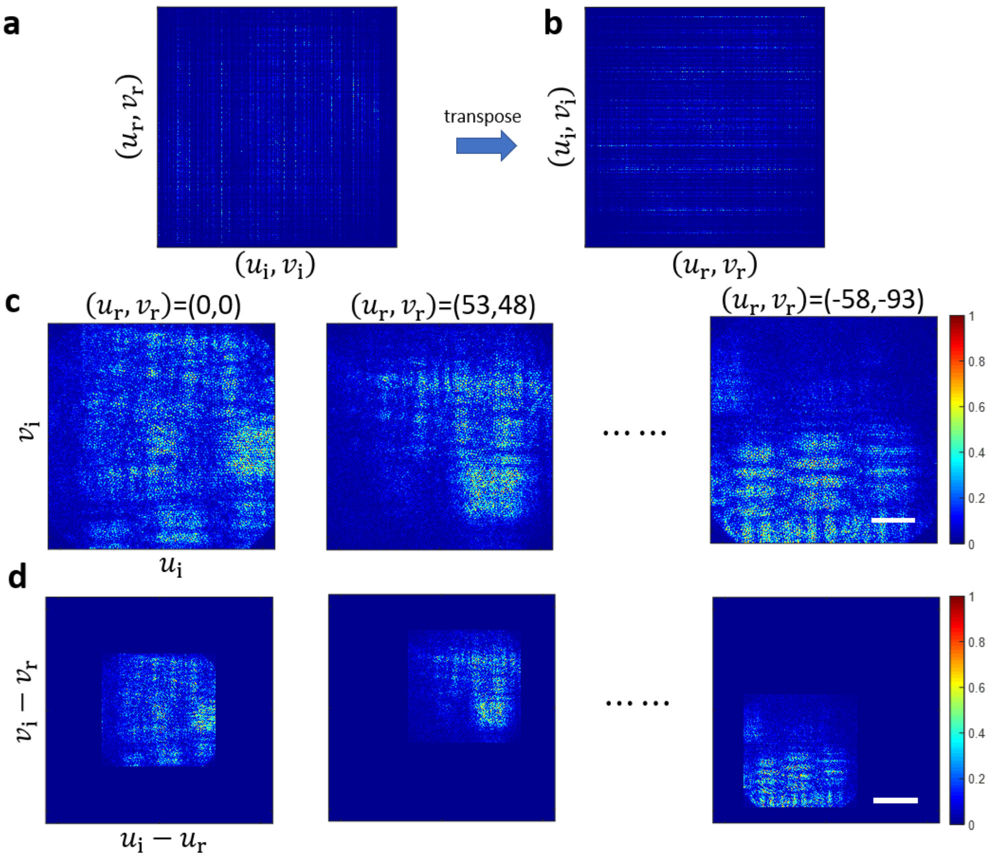

Supplementary Figure 3. Correction in the backward process. a, Reflection matrix constructed by the matrix elements $e^{-i\phi_\mathrm{i}^{(1)}(u_\mathrm{I},v_\mathrm{i})}E_\mathrm{camera}(u_\mathrm{r},v_\mathrm{r};u_\mathrm{I},v_\mathrm{i})$. **b,** Transposed reflection matrix R_{tr}. **c,** Amplitude of complex field maps $e^{-i\phi_\mathrm{i}^{(1)}(u_\mathrm{I},v_\mathrm{i})}E_\mathrm{camera}(u_\mathrm{I},v_\mathrm{I};u_\mathrm{r},v_\mathrm{r})$ obtained by individual columns of $R_{tr}^{(1)}$ in **b**. The coordinates above each figure indicate (u_r, v_r) in µm. **d,** Amplitude of complex field maps spectrally shifted by $(u_\mathrm{r}, v_\mathrm{r})$ for images in **c**. Scale bar in **c** and **d** are 50 µm and 100 µm, respectively. Colour bars in **c** and **d**, normalized amplitude.

(iii) Iteration process

Even after applying $\phi_\mathrm{i}^{(1)}$ and $\phi_\mathrm{r}^{(1)}$, the corrections are not yet complete. To increase the correction accuracy, we conducted an iteration sequence for the forward and backward correction processes. To this end, we obtain $\phi_\mathrm{i}^{(2)}$ from the correlations among the columns of $R^{(2)}$, which is the residual phase retardations in the previous round. Subsequently, we compute $\phi_\mathrm{r}^{(2)}$ from the transposed matrix of $R_{tr}^{(2)}$. We repeat the iteration process *n* times until $\phi_\mathrm{i}^{(n)}$ and $\phi_\mathrm{r}^{(n)}$ converge to a value smaller than a preset threshold value. By the addition of all the correction phases found in every iteration step, we obtain the final input phase retardation $\phi_\mathrm{i}^\mathrm{c}(u_\mathrm{i}, v_\mathrm{i}) = \sum_{j=1}^{n} \phi_\mathrm{i}^{(j)}$ and output phase retardation $\phi_\mathrm{r}^\mathrm{c}(u_\mathrm{i}, v_\mathrm{i}) = \sum_{j=1}^{n} \phi_\mathrm{r}^{(j)}$, which are shown in Fig. 2g and 2h, respectively, in the main text. To expedite the convergence, fibre dispersion found in the preceding round of the forward (backward) correction has been used as an initial condition for the next round of backward (forward) correction.

The object image is reconstructed by applying the correction with $\phi_\mathrm{i}^\mathrm{c}$ and $\phi_\mathrm{r}^\mathrm{c}$ to each of the original image

E_{camera}. And all the corrected images are properly shifted in the spatial frequency domain and then coherently combined all together in their electric fields. The reconstructed complex field map is written as

$$E_{\mathrm{recon}}(u_{\mathrm{r}}, v_{\mathrm{r}}) = \sum_{(u_{\mathrm{i}},v_{\mathrm{i}})} e^{-i\phi_{\mathrm{r}}^{\mathrm{c}}(u_{\mathrm{r}}-u_{\mathrm{i}},v_{\mathrm{r}}-v_{\mathrm{i}})} E_{\mathrm{camera}}(u_{\mathrm{r}} - u_{\mathrm{i}}, v_{\mathrm{r}} - v_{\mathrm{i}}; u_{\mathrm{i}}, v_{\mathrm{i}}) e^{-i\phi_{\mathrm{i}}^{\mathrm{c}}(u_{\mathrm{i}},v_{\mathrm{i}})}. \quad (\mathrm{S10})$$

The object image is obtained by the inverse Fourier transform of E_{recon}. Note that the intensity $|E_{\mathrm{recon}}(u_{\mathrm{r}}, v_{\mathrm{r}})|^2$ integrated over $(u_{\mathrm{r}}, v_{\mathrm{r}})$ is increased and eventually saturated as the iteration is repeated (Supplementary Figure 4) as a consequence of corrections.

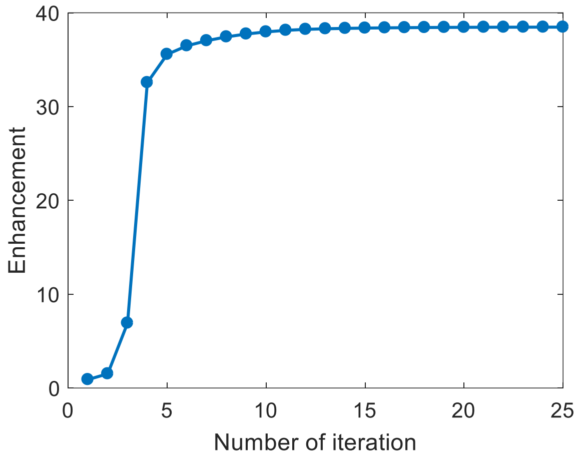

Supplementary Figure 4. Convergence curve depending on the number of iterations. The enhancement of the total intensity $\iint |E_{\mathrm{recon}}(u_{\mathrm{r}}, v_{\mathrm{r}})|^2 du_r dv_r$ was traced with the correction of an image of a USAF target in Fig. 2j. The total intensity quickly increases when the iteration starts and then saturates after about 5 iterations. The enhancement curve depends on the specific imaging conditions, but it generally saturates at about 15 iterations.

3. Resolving power and field of view

3.1. Theoretical assessment

In our lensless endomicroscopy, the complex-field map recorded by the camera $E_{\mathrm{camera}}(u_{\mathrm{r}}, v_{\mathrm{r}}; u_{\mathrm{i}}, v_{\mathrm{i}})$ corresponds to the modified object spectrum $\tilde{O}_{\mathrm{M}}\left(\frac{k}{d}(u_{\mathrm{r}} + u_{\mathrm{i}}), \frac{k}{d}(v_{\mathrm{r}} + v_{\mathrm{i}})\right)$. Thus the spatial resolution is determined by the diameter D of the field of view (FOV) of the camera and the distance d between the distal end of the fibre bundle and the sample. In the case of illuminating through the fibre core at the center, i.e. $(u_{\mathrm{i}}, v_{\mathrm{i}}) = (0,0)$, the coordinate of the spatial frequency captured by the camera is represented as $\left(\frac{k}{d}u_{\mathrm{r}}, \frac{k}{d}v_{\mathrm{r}}\right)$. Therefore, the range for the measurable spatial frequency is set by $\frac{k}{d}\sqrt{u_{\mathrm{r}}^2 + v_{\mathrm{r}}^2} \leq \frac{k}{d}(D/2)$. The numerical aperture (NA) is given by $\alpha = nD/2d$, where n is the refractive index of the immersion medium filling the space between the distal end of the fibre bundle and the sample. When the bundle diameter is increased to the extent that α exceeds the NA of the fibre core itself, which is 0.4, then the latter limits the achievable spatial resolution.

The view field diameter L of the reconstructed image is determined by the inverse of the spatial frequency resolution $\delta k/2\pi$ of the modified object spectrum $\tilde{O}_{\mathrm{M}}$ obtained by E_{camera}. δk is given by $\delta k = \frac{k}{d}\Delta D$, where ΔD is the distance between the centers of two adjacent cores. Therefore, L is given by $L = (\lambda/n)d\Delta D^{-1}$. Interestingly, we found that the effective core-to-core spacing ΔD_{eff} is approximately half the distance ΔD. This is partly because the fibre cores are randomly distributed so that the spectral resolution

along either x or y direction becomes finer than δk. The image synthesis after the spectral shift by $(u_\mathrm{i}, v_\mathrm{i})$ further reduces the effective δk. As a result, the effective view field diameter is determined as $L_\mathrm{eff} = (\lambda/n)d\Delta D_\mathrm{eff}^{-1} \cong 2L$.

Supplementary Figure 5 shows the numerical aperture α (Supplementary Figure 5a), lateral resolution (Supplementary Figure 5b), axial resolution (Supplementary Figure 5c), and L_eff (Supplementary Figure 5d) depending on d.

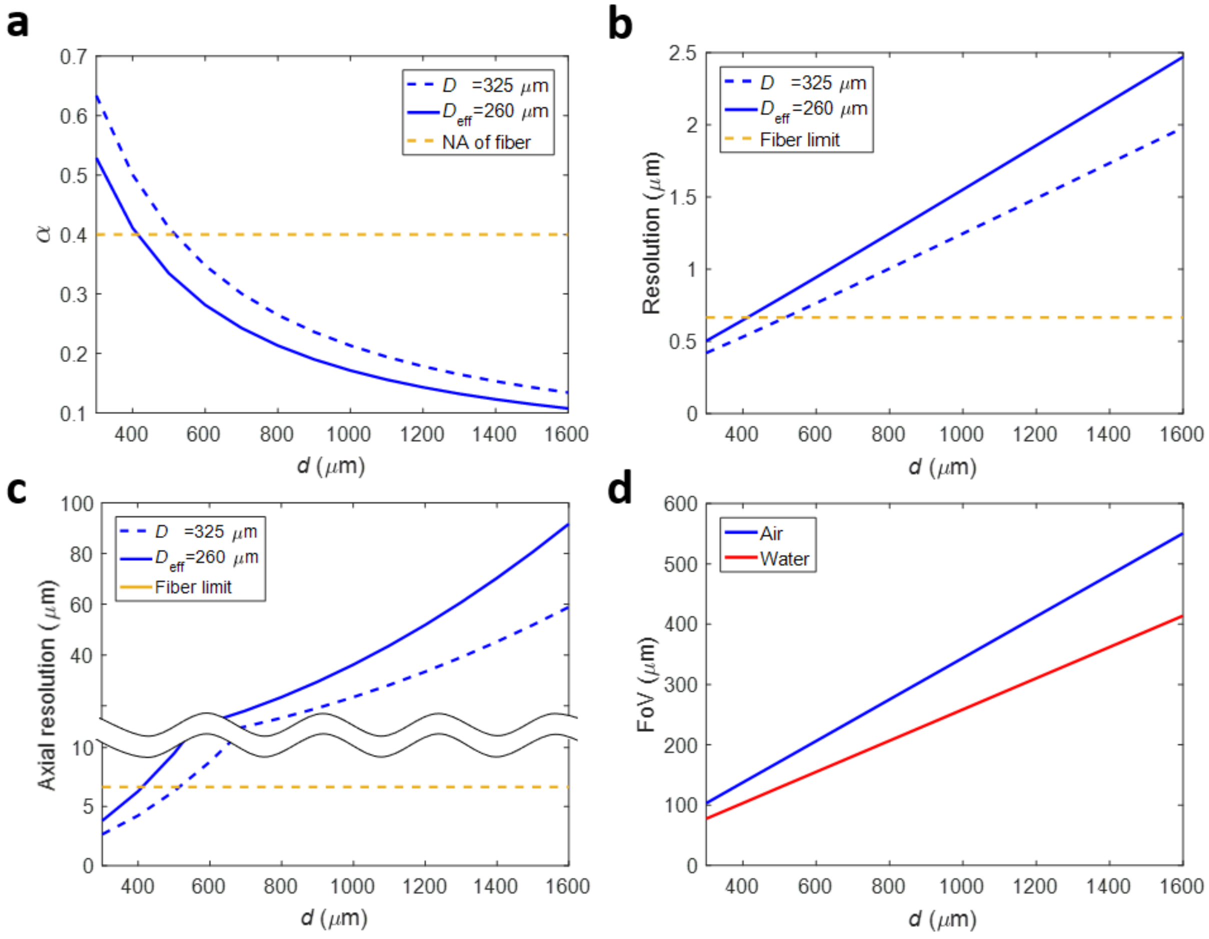

Supplementary Figure 5. The numerical aperture α, lateral resolution, axial resolution, and L_eff depending on the distance d. In **a-c**, the refractive index of the immersion medium n is 1.33. **a,** Numerical aperture α is proportional to the reciprocal of d. **b,** Lateral resolution is defined as $\frac{\lambda}{2\alpha}$, thus proportional to d. **c,** Axial resolution is defined as $2\lambda/\alpha^2$. In **a-c**, the dashed line and the solid line correspond to D and D_eff, respectively. The yellow dashed line shows the limit set by the NA of the cores in the fibre bundle. **d,** Blue and red lines show the view field diameter L_eff in air and water immersion, respectively.

Supplementary Figure 6 shows the experimentally acquired images of a USAF target depending on the distance d between the distal end of the fibre bundle and the target sample. These images were measured in the water immersion. The D_eff was $0.8D$=260 μm. With the increase of d, we could clearly observe that the view field is increased and the spatial resolving power is reduced.

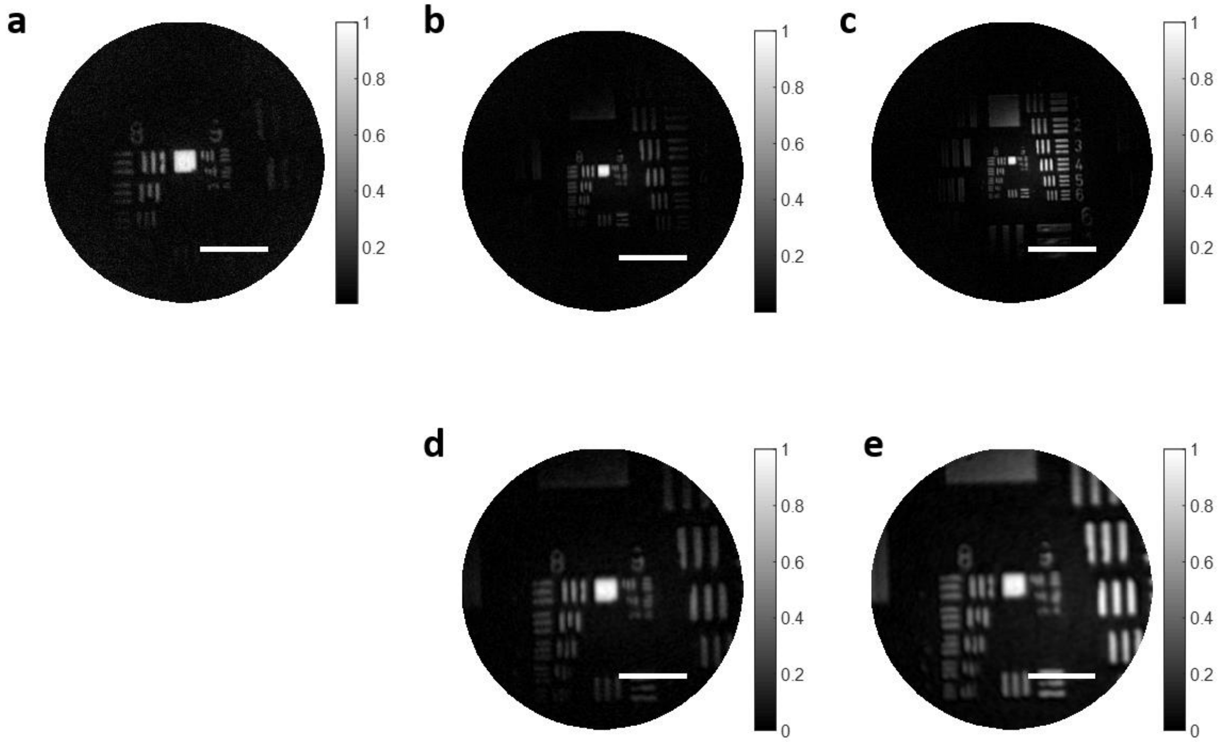

Supplementary Figure 6. The images of a USAF target depending on the distance d. All images were measured in the water immersion. In **a**-**c**, the distance d was set as 400 μm, 800 μm, and 1200 μm, respectively, and the scale bars are 25, 50, and 75 μm in order. **d** and **e** are zoomed-in images for **b** and **c**, respectively, to make their scale the same as a for the direct comparison of the spatial resolution. The scale bars in **d** and **e** are 25 μm. Element #2 in Group 9 of the USAF is clearly resolved in **a**, while it is barely resolved in **d** and unresolvable in **e**.

3.2. Lateral resolution measurements

Our endoscope could resolve the smallest feature size in the USAF 1951 target. As shown in Supplementary Figure 7a and b, the 1st element of group 9, having the distance of 1.56 μm between the neighboring bars, was clearly resolved, suggesting that lateral resolution is equal to or better than 1.56 μm. In fact, the edge of the USAF target is sharp enough to fully evaluate the endoscope resolution. Therefore, we performed the edge response function analysis (Supplementary Figure 7c). The derivative of the intensity profile along the arrow in Supplementary Figure 7a was fitted with the Gaussian function, and its FWHM was measured as 0.85 μm, which is very close to the theoretical resolution (0.78 μm) of our system.

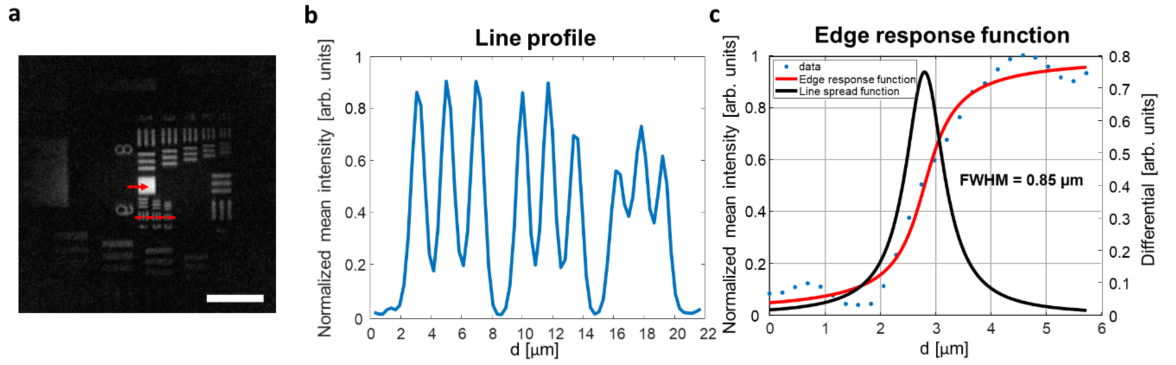

Supplementary Figure 7. Experimental evaluation of the lateral resolution. a, Fourier holographic endoscope image of a USAF target image taken at 500 µm distance from the fibre tip. Scale bar: 30 µm. **b,** Line profile along the dashed line in **a**. **c,** Edge response function analysis. Blue dots: intensity along the arrow in **a**. Red curve: fitted curve to the data by the edge response function. Black curve: derivative of the red curve. Its FWHM was measured to be 0.85 µm.

3.3. Space-bandwidth product enhancement

Our Fourier holographic endoscope eliminates the pixelation artifact of the fibre bundle, which was realized because fibre cores are sampling the spatial frequency spectra of the object. This means that there is an increase of space-bandwidth product. The enhancement estimated by (view field/resolution spot size)/(# of fibre cores in the same view field) was 13.

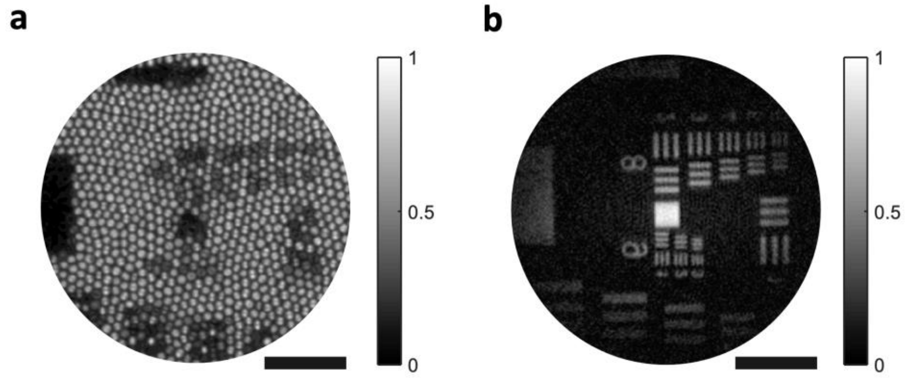

Supplementary Figure 8. Estimation of space-bandwidth product. a, Contact-mode conventional endoscope image in a transmission configuration. There are 832 fibre cores within the circular view field. **b,** Fourier holographic image with the fibre-sample distance of 500 µm. The diameter of the view field was 100 µm, and the estimated lateral resolving power was 0.85 µm, which means that the effective number of image pixels amounts to 10,865. Therefore, the space-bandwidth product was increased by 13 times. Scale bars: 30 µm.

3.4. Demonstration of volumetric imaging

We performed additional experiments validating the 3D imaging capability of our proposed method. TiO_2 particles were dispersed in agarose gel, and a set of raw images were taken at a given position of the fibre bundle. Our algorithm initially reconstructed particles at the depth of 600 µm (Supplementary Figure 9).

We then performed the numerical propagation to obtain images at other depths. It can be seen that different particles appeared as the depth of reconstruction varied (see details in the Supplementary Movie 2). This confirms the volumetric imaging capability of the proposed method.

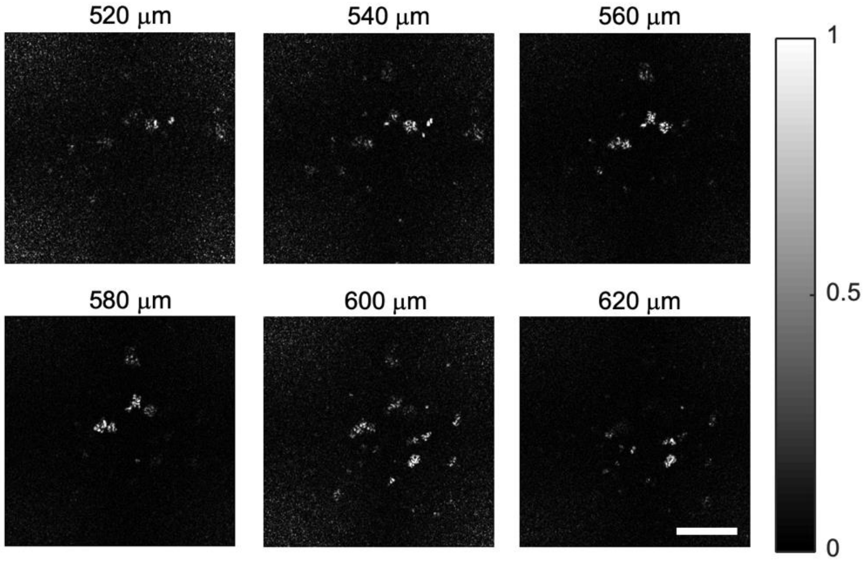

Supplementary Figure 9. Volumetric imaging of TiO_2 particles embedded in an agarose gel. Object plane distance from the fibre distal tip was indicated above each figure panel. Scale bar: 40 μm.

3.5. Depth resolution measurements

We performed numerical propagation of the images of TiO_2 particles reconstructed at tip-sample distance of 540 μm (Supplementary Figure 10a) which were shown in Supplementary Figure 9. From the axial profile of a particle marked by a yellow arrow, we could obtain a depth resolution of 14 μm (Supplementary Figure 10b). This was slightly worse than the theoretical expectation estimated by the fibre numerical aperture (10.5 μm axial resolution at 540 μm distance). This was in part due to the decreased collection efficiency at cores farther away from the center of the fibre bundle, which led to the decrease of effective numerical aperture (NA). Considering that axial resolution scales with $1/NA^2$, effective NA is estimated to be a factor 0.87 of the ideal case.

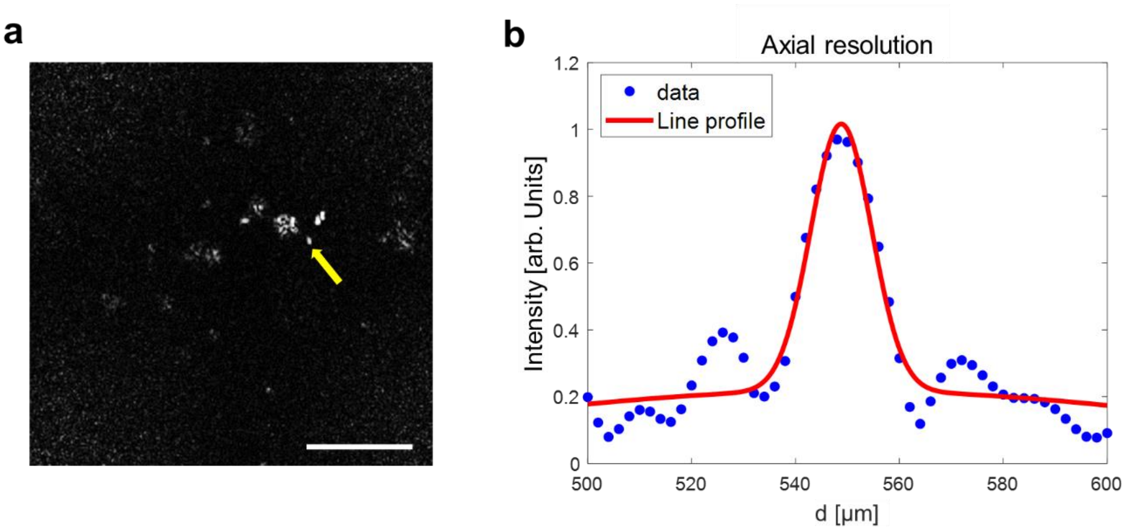

Supplementary Figure 10. Measurement of the axial resolution. a, Image of TiO_2 particles reconstructed when the distal tip of the fibre bundle was 540 μm away for the target. Scale bar, 40 μm. **b,** Image in **a** was numerically propagated, and the intensity at a particle marked by the yellow arrow was measured as a function of the propagation distance d.

4. Minimum working distance

In Supplementary Figure 11, we showed reconstructed images taken at various distances. The reconstruction fidelity was high down to $d = 400\ \mu m$. However, further reduction in d led to the gradual decrease of SNR. The effective area where target structures were reconstructed was about 20 μm in diameter at $d = 300\ \mu m$, and it was reduced further at $d = 200\ \mu m$. At $d = 150\ \mu m$, the reconstruction fidelity was too low to discern any structures.

As the distance d is reduced, the validity of the Fresnel approximation is gradually violated. While the condition, $kd\frac{1}{8}\left[\left(\frac{x-u_\mathrm{i}}{d}\right)^2 + \left(\frac{y-v_\mathrm{i}}{d}\right)^2\right]^2 \ll 1$ should be satisfied in the strict sense, the Fresnel approximation can be valid when $\sqrt{|x-u_\mathrm{i}|^2 + |y-v_\mathrm{i}|^2} \leq 2\sqrt{\lambda d}$ in an optimistic condition (Fourier optics, Goodman Chapter 4). Here, $(u_\mathrm{i}, v_\mathrm{i})$ and (x, y) indicate spatial coordinates of illumination core and object plane, respectively. For example, at $d = 300\ \mu m$, a circular area at the sample plane (x, y) with a radius of 24 μm satisfies the approximation with respect to the core at $(u_\mathrm{i}, v_\mathrm{i})$. Since the propagation from an object plane (x, y) to a detection core $(u_\mathrm{r}, v_\mathrm{r})$ poses a similar constraint, the area in the sample that meets the Fresnel approximation will be smaller. Considering these factors, the experimentally observed effective area goes well with the valid range set by the Fresnel approximation. When the valid area is too small, the correlation among columns of the reflection matrix lower, and signal from the rest of the area contributes to the noise. This led to the breakdown of the reconstruction algorithm, as is the case for $d = 150\ \mu m$.

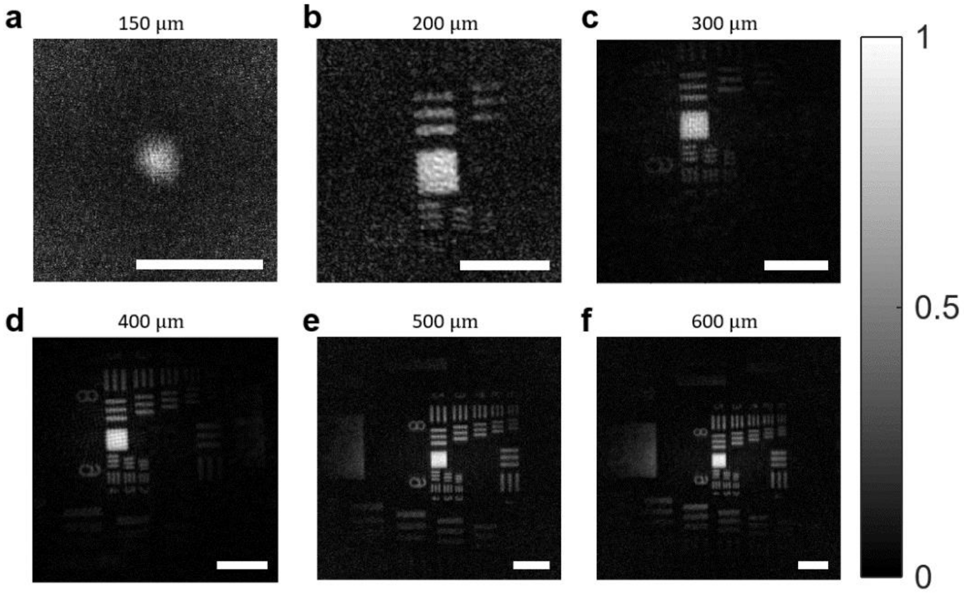

Supplementary Figure 11. USAF target images taken at various fibre-sample distances indicated above individual figure panels. Scale bars: 20 μm.

Our reconstruction algorithm makes use of the fact that the modified object function, $O_{\mathrm{M}}(x,y) = O(x,y)exp\left\{i\frac{k}{d}(x^2+y^2)\right\}$ is invariant with respect to the choice of illumination cores under the Fresnel approximation condition. More specifically, the detected wave at the camera is given as Eq. (S6). Aside from the phase retardations, $\phi_{\mathrm{i}}(u_{\mathrm{i}},v_{\mathrm{i}})$ and $\phi_{\mathrm{r}}(u_{\mathrm{r}},v_{\mathrm{r}})$, induced by the fibre bundle, $E_{\mathrm{camera}}(u_{\mathrm{r}},v_{\mathrm{r}};u_{\mathrm{i}},v_{\mathrm{i}})$ is shift-invariant with respect to the choice of illumination core $(u_{\mathrm{i}},v_{\mathrm{i}})$. Therefore, normalized correlation between $E_{\mathrm{camera}}(u_{\mathrm{r}},v_{\mathrm{r}};0{,}0)$ and $E_{\mathrm{camera}}(u_{\mathrm{r}}-u_{\mathrm{i}},v_{\mathrm{r}}-v_{\mathrm{i}};u_{\mathrm{i}},v_{\mathrm{i}})$ should be unity with the increase of the distance of the illumination core $r=\sqrt{u_{\mathrm{i}}^2+v_{\mathrm{i}}^2}$ from the center of the fibre bundle at $(u_{\mathrm{i}},v_{\mathrm{i}})=(0{,}0)$. In the regime where Fresnel approximation is not fully valid, the normalized correlation is expected to be decreased.

To check the range where shift-invariance is valid, we conducted numerical simulation and calculated $E_{\mathrm{camera}}(u_{\mathrm{r}},v_{\mathrm{r}};u_{\mathrm{i}},v_{\mathrm{i}})$ at the same configuration as the experiment. In computing the wave propagation from fibre bundle to an object and from the object to the fibre bundle, we employed either Fresnel approximation or exact angular spectrum method. As shown in Supplementary Figure 12, the correlation stays unity with the increase of r in the case of Fresnel approximation. In the case of using exact angular spectrum method, the correlation decreases with increase of r. Up to the distance of 500 μm, the correlation persists quite a long distance beyond 60 μm. However, as d decreases below 400 μm, the persistent distance decreases to 20 μm or less. Therefore, the validity of Fresnel approximation is limited around d=400 μm, which goes well with the experimental results in Supplementary Figure 11.

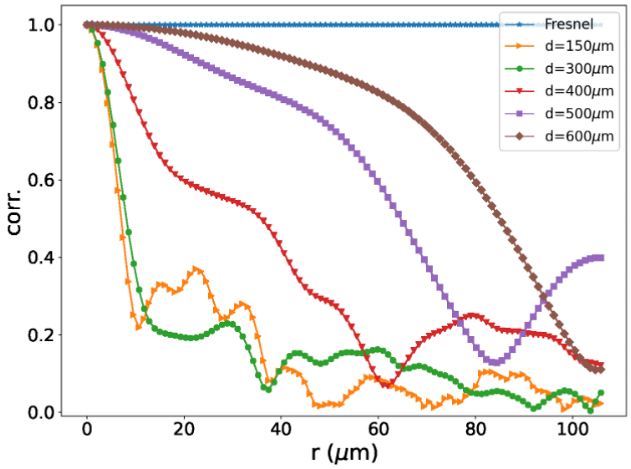

Supplementary Figure 12. Normalized correlation between $E_{\text{camera}}(u_{\text{r}}, v_{\text{r}}; 0, 0)$ and $E_{\text{camera}}(u_{\text{r}} - u_{\text{i}}, v_{\text{r}} - v_{\text{i}}; u_{\text{i}}, v_{\text{i}})$ as a function of $r = \sqrt{u_{\text{i}}^2 + v_{\text{i}}^2}$.

5. The number of images required for image reconstruction

The number of images required for image reconstruction is the main factor limiting the overall image acquisition speed. The less, the faster. To investigate the minimum number of images required for the reconstruction, we started with the image in Supplementary Figure 6c where 3000 raw images were used for reconstruction. We repeated the reconstruction after reducing the number of raw images. We considered two ways to reduce the number of images. First, the subsets of images with the reduced numbers were constructed by sparsely, but uniformly resampling the measured set of images. Supplementary Figure 13b to 13d show the reconstruction results with 1000, 200, and 100 images in order. In this method, the density of the spiral scanning (shown in Fig. 2a in the main text) was reduced while keeping its maximum radius to be D_{eff}. Therefore, spatial resolution was maintained while the image fidelity was reduced. Second, the subsets of raw images were chosen by taking only the first parts of images starting from the center of the fibre bundle. Supplementary Figure 13e to 13g show the reconstruction results with the subset of 1000, 200, and 100 images. In the second method, the maximum radius of the spiral scanning was reduced accordingly while maintaining the sampling density the same. Therefore, a reduction in the spatial resolution is expected. When comparing Supplementary Figure 13d and 13g, which are reconstructions from 100 images, to Supplementary Figure 13a, the reconstruction from the entire set of images, it was found that using only 100 images is enough to reconstruct the image with reasonable quality, especially for the central region. Also comparing Supplementary Figure 13d and 13g, it is seen that the background region in Supplementary Figure 13g is clearer than Supplementary Figure 13d.

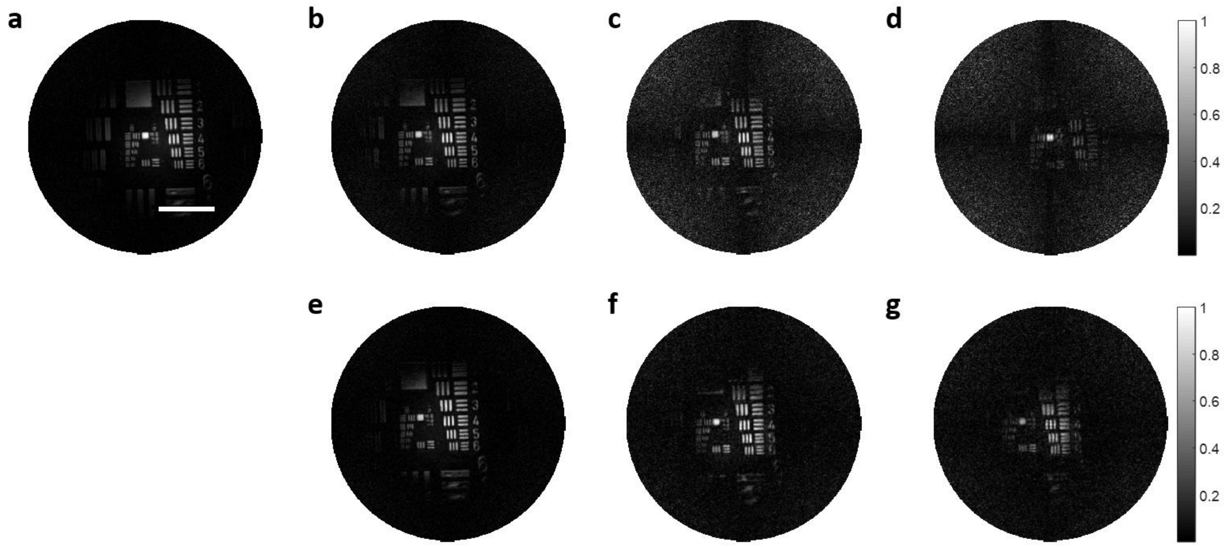

Supplementary Figure 13. Reconstructed image quality depending on the number of images. a, Reconstructed image using the entire set of 3000 images. This is the same as that in Supplementary Figure 6c. **b-d,** Reconstructed images using the subsets constructed by the sparse samplings, where images were chosen at every 3, 15, and 30 images from the entire set. The number of images in each set is 1000, 200, and 100 in order. **e-g,** Reconstructed images using the subsets created by selecting the first 1000, 200, and 100 images, in order, from the entire set. In **d** and **g**, only 100 images were used for the reconstructions. The image in **g** has a better signal to background ratio (SBR) than that in **d**. The scale bar is 75 μm in **a**, and all the images are drawn on the same scale.

We provided the effect of the number of raw images (N) to the image quality. The major benefits of using a larger number of images are the increase of SBR and SNR. As shown in Supplementary Figure 14a, we chose two areas marked by the red and blue square boxes, representing the sample and background areas, respectively. SBR was estimated by the ratio of their average intensities, and SNR was obtained by the average intensity of the red square area divided by the standard deviation in the blue rectangular area. As shown in Supplementary Figure 14b, both the SBR and SNR increase up to N=2,500. SNR at N=2,500 was approximately twice larger than that at N=200. Further increase in N resulted in the reduction of SNR and SBR. This is mainly because raw images taken by illuminating cores far from the center of the fibre bundle contain less signal.

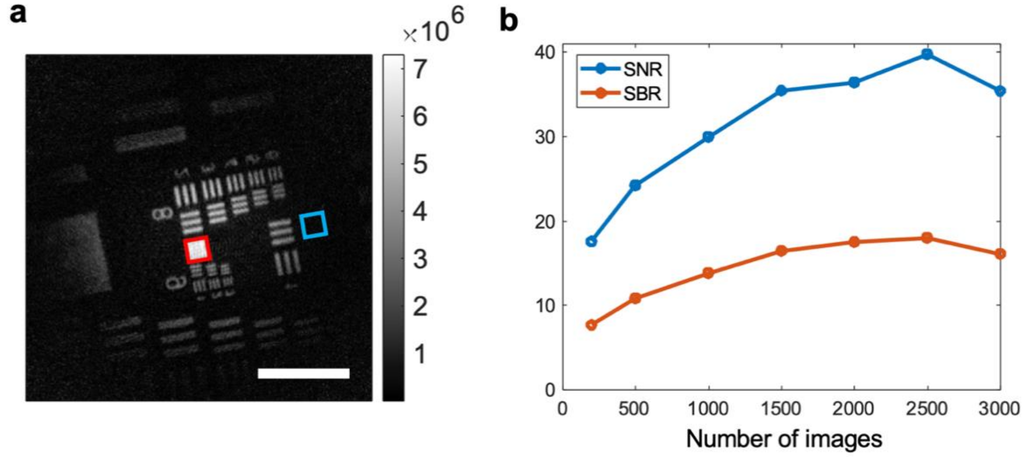

Supplementary Figure 14. SBR and SNR depending on the number of raw images. a, Representative reconstructed image of a USAF target taken for fibre-sample distance of 600 μm, and the number of images *N*=1,000. Scale bar: 40 μm. **b,** Signal-to-noise ratio (SNR) and signal-to-background ratio (SBR) estimated based on the intensity maps in red and blue rectangular boxes in **a** as a function of the number of images *N*.

6. Imaging with a 200-μm-diameter fibre bundle

A 350-μm-diameter fibre bundle was used for the endoscopic imaging of the USAF target and rat intestine tissues. Here, we showed the endoscopic imaging using a 200-μm-diameter fibre bundle (Supplementary Figure 15a). We set the standoff distance to be 800 μm in water immersion for the direct comparison with the results in Supplementary Figure 6b. Resolution target image was faithfully reconstructed even with this thinner fibre bundle. As a trade-off, the resolving power was somewhat reduced due to the reduction in diameter, and the element #2 in Group 9 of the USAF is no longer visible. D_{eff} for the 350-μm-diameter fibre bundle was 280 μm, but D_{eff} for the 200-μm-diameter fibre bundle was 200 μm. Therefore, the numerical aperture α was reduced from 0.23 to 0.17. In Supplementary Figure 15b, we showed the image reconstruction of the 350-μm-diameter fibre bundle data after computationally cropping the raw images to reduce D_{eff} to 200 μm. The resolving power was almost identical to Supplementary Figure 15a taken by the 200-μm-diameter fibre bundle, suggesting that the reduction in D_{eff} is the main cause of resolving power reduction.

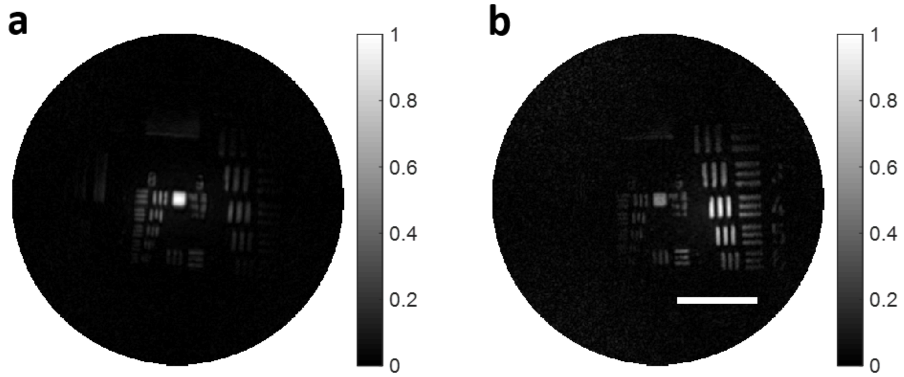

Supplementary Figure 15. Reconstructed image measured by a thinner fibre bundle. a, Reconstructed image using the 200-μm-diameter fibre bundle with standoff distance 800 μm in water immersion. D_{eff} is 200 μm. **b,** Reconstructed image of the 300-μm-diameter fibre bundle data in Supplementary Figure 6b cropping raw images to reduce D_{eff} to 200 μm. Scale bar, 50 μm.

7. Endoscopic imaging with various bending configurations

With the capability of correcting the core-to-core phase retardations in situ, we demonstrated that our endoscopic imaging method works for arbitrary bending configurations of the fibre bundle. For this purpose, we placed three different resolution targets at different positions as schematically shown in Supplementary Figure 16a and then measured $E_{\mathrm{camera}}(u_{\mathrm{r}}, v_{\mathrm{r}}; u_{\mathrm{i}}, v_{\mathrm{i}})$ for each target by navigating the end of the endoscopic probe. This was accompanied by the inevitable change of the bending configuration of the fibre bundle as drawn in Supplementary Figure 16a. Objects I and III were USAF resolution targets, and object II was a Siemens star-like target. The distance between the neighboring targets was about 12 cm. Supplementary

Figure 16b to S16d show the reconstructed images for the three objects after correcting the bending-induced phase retardations, and Supplementary Figure 16e to 16g present $\phi_{\mathrm{r}}(u_{\mathrm{r}}, v_{\mathrm{r}})$ for the respective bending configurations. The correlations between the phase maps were about 0.15, implying that the bending-induced phase retardations were almost decorrelated while the fibre probe was navigated. These results confirmed that our method realizes fully flexible endoscopy.

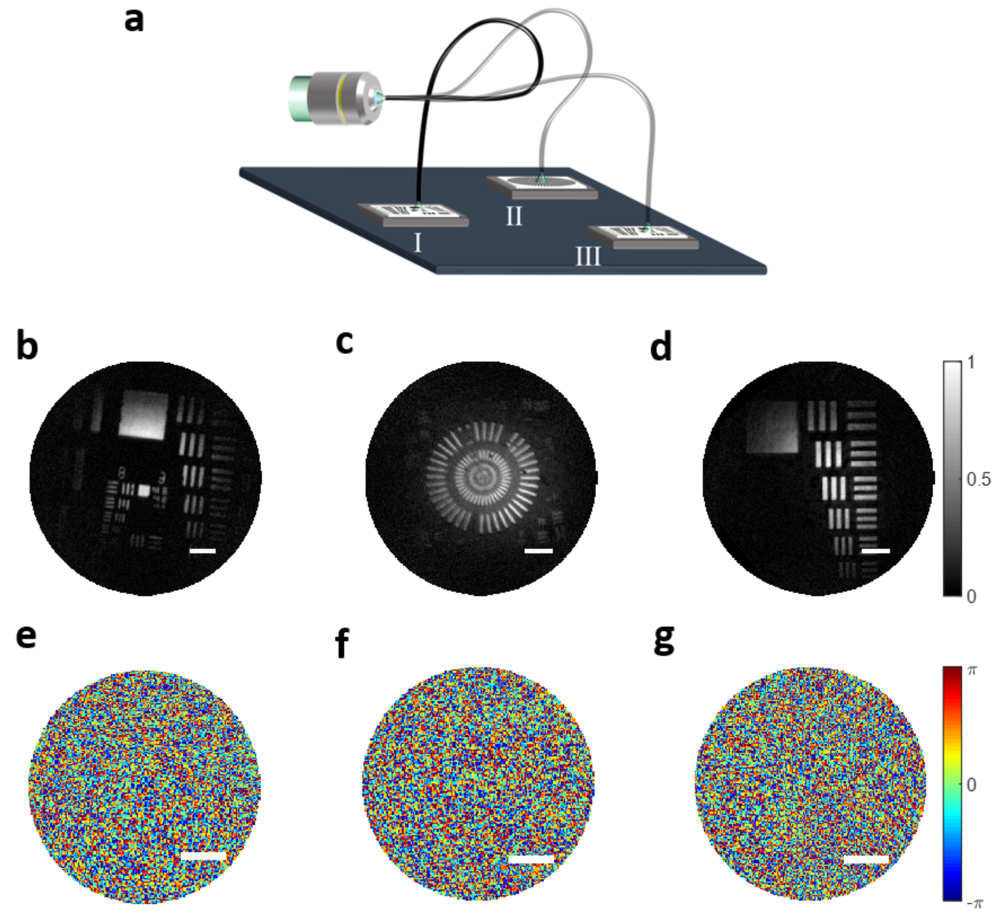

Supplementary Figure 16. Reflectance endoscopic imaging at arbitrary bending configurations of the fibre probe. a, Endoscope probe was navigated to three different samples about 12 cm apart from one another. The length of the fibre bundle was 1 m, and the detection objective lens at the proximal side was fixed. **b-d,** Reconstructed images for targets I, II, and III shown in **a**. Scale bar, 20 μm. Colour bar, normalized amplitude. **e-g,** Bending-induced phase retardations $\phi_{\mathrm{r}}(u_{\mathrm{r}}, v_{\mathrm{r}})$ used for the reconstructions in **b** to **d**. Scale bar, $0.1k_0$. Colour bar, phase in radians.

8. 20 Hz endoscopic imaging using a high-speed CMOS camera

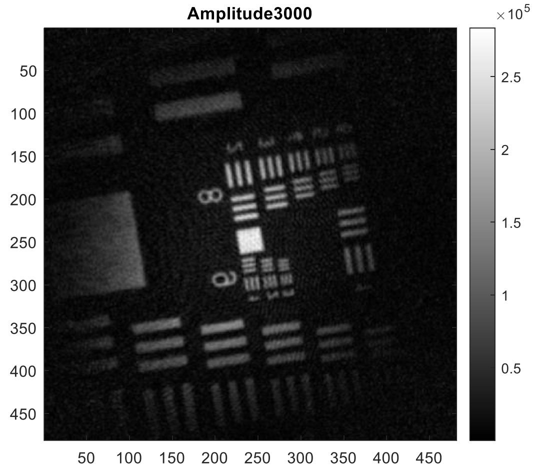

Supplementary Figure 17. 20 Hz endoscopic imaging using a high-speed CMOS camera. The sCMOS camera was replaced with a CMOS camera (Photron, FASTCAM mini UX100). Since the pixel size of this fast camera (10 μm) was different from that of sCMOS camera (6.5 μm), we switched the diffraction grating in the reference beam path with a smaller pitch for optimal interferometric image acquisition. 100 raw images were acquired at a frame rate of 2,000 Hz while scanning the illumination core using a galvanometer scanning mirror. The scanning mirror and image acquisition were synchronized during the image acquisition.
