# Supplementary material for: Flexible-type ultrathin holographic endoscope for microscopic imaging of unstained biological tissues
Source: Nat Commun. 2022 Aug 2;13:4469. doi: 10.1038/s41467-022-32114-5 (PMC9345988; doi:10.1038/s41467-022-32114-5)
Supplement: Supplementary file 3 — Description of Additional Supplementary Files [file 41467_2022_32114_MOESM3_ESM.docx]

**Supplementary Movie 1.** 3D image reconstruction from a single matrix recording for the stacked targets in Fig. 3 in the main text. After the object shape at 600 μm (depth I in Fig. 3 in the main text) was retrieved by the reconstruction algorithm, all the image information ranging from 450 μm to 1300 μm was generated by the numerical refocusing method. At 1060 μm, the second structures (depth II in Fig. 3 in the main text) were shown up clearly.

**Supplementary Movie 2.** Volumetric image reconstruction from a single matrix recording for the TiO_2_ particles embedded in an agarose gel in Fig. S9 in the Supplementary Information. The particle distribution at 600 μm was first retrieved by the reconstruction algorithm and all the image information ranging from 500 μm to 610 μm was generated by the numerical refocusing method. The axial resolution of our endoscope was determined from the axial distribution of a single particle.
