# Supplementary material for: Flexible-type ultrathin holographic endoscope for microscopic imaging of unstained biological tissues
Source: Nat Commun. 2022 Aug 2;13:4469. doi: 10.1038/s41467-022-32114-5 (PMC9345988; doi:10.1038/s41467-022-32114-5)
Supplement: Supplementary file 6 — Reporting Summary [file 41467_2022_32114_MOESM6_ESM.pdf]

Corresponding author(s): Youngwoon Choi, Wonshik Choi

Last updated by author(s): May 24, 2022

Reporting Summary

Nature Portfolio wishes to improve the reproducibility of the work that we publish. This form provides structure for consistency and transparency in reporting. For further information on Nature Portfolio policies, see our Editorial Policies and the Editorial Policy Checklist.

Statistics

For all statistical analyses, confirm that the following items are present in the figure legend, table legend, main text, or Methods section.

n/a	Confirmed	
☐	☒	The exact sample size (n) for each experimental group/condition, given as a discrete number and unit of measurement
☐	☒	A statement on whether measurements were taken from distinct samples or whether the same sample was measured repeatedly
☒	☐	The statistical test(s) used AND whether they are one- or two-sided<br>*Only common tests should be described solely by name; describe more complex techniques in the Methods section.*
☒	☐	A description of all covariates tested
☒	☐	A description of any assumptions or corrections, such as tests of normality and adjustment for multiple comparisons
☒	☐	A full description of the statistical parameters including central tendency (e.g. means) or other basic estimates (e.g. regression coefficient) AND variation (e.g. standard deviation) or associated estimates of uncertainty (e.g. confidence intervals)
☒	☐	For null hypothesis testing, the test statistic (e.g. F, t, r) with confidence intervals, effect sizes, degrees of freedom and P value noted<br>*Give P values as exact values whenever suitable.*
☒	☐	For Bayesian analysis, information on the choice of priors and Markov chain Monte Carlo settings
☒	☐	For hierarchical and complex designs, identification of the appropriate level for tests and full reporting of outcomes
☒	☐	Estimates of effect sizes (e.g. Cohen's d, Pearson's r), indicating how they were calculated

Our web collection on statistics for biologists contains articles on many of the points above.

Software and code

Policy information about availability of computer code

Data collection	Custom codes built with MATLAB (R2020b) were used to control the instruments including the sCMOS camera and galvanometer mirrors. The MATLAB codes are not central to the research. The MATLAB codes used in this work are available from the corresponding authors upon request.
Data analysis	Custom codes built with MATLAB (R2020b) were used to convert the acquired images into datasets. As we used common command lines, our MATLAB codes are not central to the research. The MATLAB codes used in this work are available from the corresponding authors upon request.

For manuscripts utilizing custom algorithms or software that are central to the research but not yet described in published literature, software must be made available to editors and reviewers. We strongly encourage code deposition in a community repository (e.g. GitHub). See the Nature Portfolio guidelines for submitting code & software for further information.

Data

Policy information about availability of data

All manuscripts must include a data availability statement. This statement should provide the following information, where applicable:

- Accession codes, unique identifiers, or web links for publicly available datasets
- A description of any restrictions on data availability
- For clinical datasets or third party data, please ensure that the statement adheres to our policy

The datasets acquired for this study are available from the corresponding authors upon request. The size of the datasets are too large to deposit on the publicly

accessible repository.

Human research participants

Policy information about studies involving human research participants and Sex and Gender in Research.

Reporting on sex and gender	This is not relevant to our study.
Population characteristics	This is not relevant to our study.
Recruitment	This is not relevant to our study.
Ethics oversight	This is not relevant to our study.

Note that full information on the approval of the study protocol must also be provided in the manuscript.

Field-specific reporting

Please select the one below that is the best fit for your research. If you are not sure, read the appropriate sections before making your selection.

- [x] Life sciences
- [ ] Behavioural & social sciences
- [ ] Ecological, evolutionary & environmental sciences

For a reference copy of the document with all sections, see nature.com/documents/nr-reporting-summary-flat.pdf

Life sciences study design

All studies must disclose on these points even when the disclosure is negative.

Sample size	We used 5 of 1- to 2-day-old Sprague-Dawley (SD) rats.
Data exclusions	No data were excluded from the analysis.
Replication	We performed imaging 1- to 2-day-old SD rats 5 times and obtained similar results for all attempts. The best result was presented.
Randomization	This is not relevant to our study.
Blinding	This is not relevant to our study.

Reporting for specific materials, systems and methods

We require information from authors about some types of materials, experimental systems and methods used in many studies. Here, indicate whether each material, system or method listed is relevant to your study. If you are not sure if a list item applies to your research, read the appropriate section before selecting a response.

Materials & experimental systems

n/a	Involved in the study	
x		Antibodies
x		Eukaryotic cell lines
x		Palaeontology and archaeology
	x	Animals and other organisms
x		Clinical data
x		Dual use research of concern

Methods

n/a	Involved in the study	
x		ChIP-seq
x		Flow cytometry
x		MRI-based neuroimaging

Animals and other research organisms

Policy information about studies involving animals; ARRIVE guidelines recommended for reporting animal research, and Sex and Gender in Research

Laboratory animals	We used 1- to 2-day-old Sprague-Dawley rats.
Wild animals	This study did not involve wild animals.

Reporting on sex	Both male and female SD rats were used.
Field-collected samples	This study did not involve samples collected from the field.
Ethics oversight	All of the experimental procedures and protocols above were conducted in accordance with the guidelines established by the Committee of the Animal Research Policy at Korea University.

Note that full information on the approval of the study protocol must also be provided in the manuscript.
