# Supplementary material for: Flexible-type ultrathin holographic endoscope for microscopic imaging of unstained biological tissues
Source: Nat Commun. 2022 Aug 2;13:4469. doi: 10.1038/s41467-022-32114-5 (PMC9345988; doi:10.1038/s41467-022-32114-5)
Supplement: Supplementary file 2 — Peer Review File [file 41467_2022_32114_MOESM2_ESM.pdf]

nature portfolio

Peer Review File

Flexible-type ultrathin holographic endoscope for microscopic imaging of unstained biological tissues

Open Access This file is licensed under a Creative Commons Attribution 4.0 International License, which permits use, sharing, adaptation, distribution and reproduction in any medium or format, as long as you give appropriate credit to the original author(s) and the source, provide a link to the Creative Commons license, and indicate if changes were made. In the cases where the authors are anonymous, such as is the case for the reports of anonymous peer reviewers, author attribution should be to 'Anonymous Referee' followed by a clear attribution to the source work. The images or other third party material in this file are included in the article's Creative Commons license, unless indicated otherwise in a credit line to the material. If material is not included in the article's Creative Commons license and your intended use is not permitted by statutory regulation or exceeds the permitted use, you will need to obtain permission directly from the copyright holder. To view a copy of this license, visit http://creativecommons.org/licenses/by/4.0/.

Editorial Note: This manuscript has been previously reviewed at another journal that is not operating a transparent peer review scheme. This document only contains reviewer comments and rebuttal letters for versions considered at *Nature Communications*.

REVIEWERS' COMMENTS

Reviewer #2 (Remarks to the Author):

The authors have sufficiently addressed the referee comments and made substantial improvements to their manuscript.

Reviewer #4 (Remarks to the Author):

I have reviewed the comments by the two previous reviewers and the response from the authors.

I have found that the authors provided significant additional work that validated and even bettered their initial work.

I found it clear and well executed as well as impressive. It is the first real time high resolution endoscope with corrections for fiber bending and thus deserves publication.
